# Influence of TiN Coating on the Drawing Force and Friction Coefficient in the Deep Drawing Process of AlMg4.5Mn0.7 Thin Sheets

**DOI:** 10.3390/ma16113968

**Published:** 2023-05-25

**Authors:** Milan T. Djordjević, Srbislav Aleksandrović, Dušan Arsić, Ružica R. Nikolić, Janusz Szmidla, Aleksandar Todić, Dragan Čukanović, Robert Ulewicz

**Affiliations:** 1 Faculty of Technical Sciences, University of Pristina, 38220 Kosovska Mitrovica, Serbia; milan.t.djordjevic@pr.ac.rs (M.T.D.);; 2 Faculty of Engineering, University of Kragujevac, 34000 Kragujevac, Serbia; srba@kg.ac.rs; 3 Research Centre, University of Zilina, 01026 Zilina, Slovakia; 4 Department of Mechanics and Machine Design Fundamentals, Czestochowa University of Technology, 42-201 Czestochowa, Poland; 5 Department of Production Engineering and Safety, Czestochowa University of Technology, 42-201 Czestochowa, Poland

**Keywords:** Al alloy, TiN coating, flat die deep drawing process, contact pressure, coefficient of friction

## Abstract

The influence of various process parameters on the deep drawing process is a current research topic in sheet metal forming technology. Starting from the application of the previously constructed original testing device, an original tribological model was developed based on the process of sheet metal strip sliding between flat contact surfaces under variable pressures. A complex experiment was executed using an Al alloy sheet, tool contact surfaces of different roughness, two types of lubricants and variable contact pressures. The procedure included analytically pre-defined contact pressure functions based on which, for each of the mentioned conditions, the dependencies of the drawing forces and friction coefficients were obtained. The pressure in function P1 constantly decreased from a high initial value until the minimum, while in function P3 the pressure increased until the minimum value at the halfway point of the stroke, after which it increased up to the initial value. On the other hand, the pressure in function P2 constantly increased from the initial minimum value until the maximum value, while in function P4 the pressure increased until reaching the maximum value at the halfway point of the stroke, after which it decreased to the minimum value. This enabled the determination of the influence of tribological factors on the process parameters of intensity of traction (deformation force) and coefficient of friction. The pressure functions starting with decreasing trends produced higher values for the traction forces and the friction coefficient. In addition, it was established that the roughness of the contact surfaces of the tool, especially those with titanium nitride coating, has a significant influence on the process parameters. For surfaces of lower roughness (polished), a tendency of the Al thin sheet to form a glued-on layer was noticed. This was especially prominent for lubrication with MoS_2_-based grease under conditions of high contact pressure (functions P1 and P4 at the beginning of the contact).

## 1. Introduction

There are only a few factors that influence the deep drawing process—the effect of contact pressure on the thin sheet flange and the action of the draw beads at the point of contact with holder. In most of the previous research on this topic, the pressure within a die was considered (or set) as constant. In this research, however, the focus is on variable pressure. This was achieved by continuous pressure setting during the sliding process via theoretically pre-set functions of pressure variations in terms of time. Thus, the influence of variable contact pressure was the actual subject of this research for the purpose of defining yet another factor to control the forming process. The other influential factors (the die, the contact conditions or material) were not considered.

Out of the many developed physical–tribological models of the forming process, the most-studied one is the flat die sliding model [1,2,3,4,5]. The authors of the relevant papers considered models of the deep drawing process using a thin sheet flange at the flat contact surfaces between the holder and a die. The created tribological models took into account all the influential factors (material, die, contact conditions) to be able to monitor variations in the friction coefficient and the drawing force with the application of tools of various surface roughness. The contact conditions were realized with the use of various types of deep drawing lubricant and thin sheets with different coatings. In some experiments, the authors reported variation in the thin sheet sliding speed as well [6,7,8]. The objective was always how to control the output process parameters in order to reduce the friction coefficient and drawing forces (as much as possible), while simultaneously obtaining the desired geometry of the forming product without wrinkles at the flange [9,10,11].

This is why, in this paper, we tried to consider several factors influencing the deep drawing process. The original tribological model was described using Al alloy sheets with surfaces of variable roughness, with contact pressure variation in terms of time according to four preset functions and with the application of two different lubricants. The considered output parameters were the traction force and the friction coefficient.

## 2. Literature Review

The presented review of some of the mentioned references follows the two major “laws” in scientific research, the results of which are to be usefully and successfully applicable in practice.

“Review of the literature indicates that the choice of the testing technique, the construction of the apparatus and close control of the experimental variables, are the most important in developing consistent data that are representative of industrial conditions” [12].

“The ultimate aim of applied research is generation of knowledge relevant to production processes. A vital first step in acquiring such knowledge is the choice of experimental methodology” [13].

An experimental evaluation of the friction coefficient during thin sheet strip sliding is presented by Frattini et al. (2006) in [1]. The authors developed a simple measurement system that aimed to reproduce the process conditions occurring during the typical sheet metal stamping operation. It recorded the force variation on the specimen with the set contact conditions. The used samples included cylindrical dies and strips from different sheets, with or without coating. The obtained results were sufficiently reliable to be used in the forming processes of thin sheets with similar sliding conditions.

Szakaly and Lenard (2010) reported the application of a different apparatus, more massively built; this massiveness was explained by an intention to minimize the dispersion of the test results [2]. Their results confirmed that at higher sliding speeds and higher contact pressures, the friction coefficient values decreased. Higher die roughness did not always guarantee higher values for the friction coefficient.

Figueiredo et al. (2011) investigated thin sheet friction effects using two different techniques to assess friction [3]. The obtained results revealed that using the cross-sliding test, one can obtain the reduced friction, which was attributed to slightly increased contact pressure. The friction coefficient decreased with the number of realized slidings, which was probably caused by the surfaces’ running-in effect.

Coello et al. (2013) studied sliding between the flat surfaces of thin sheets made of high-strength steel and coated with zinc [4]. The roughness of the contact surfaces was in the form of asperities, which cause the creation and retaining of micro-pockets of lubricant. This resulted in more favorable friction conditions. Different lubrication regimes may be present in a sliding system. Furthermore, certain lubrication regimes could vary during the forming process. Thus, the sheets could be subjected to different tribological conditions in different process stages. The friction coefficient dropped with increases in sliding speed and contact pressure. The applied lubricant layer’s thickness had no effect on the test results.

Yanagida and Azushima (2019) considered the influences of lubricant, temperature and contact pressure on the friction coefficient [5]. They used a tribo-simulator in dry conditions. The obtained coefficients of friction were applicable for use in numerical simulations with finite element analysis.

Manoylov et al. (2013) studied the elasto-plastic contact of nominally plane parallel surfaces [6]. The local separation of surfaces is significantly influenced by surface roughness. In the mixed lubrication, the lubricant film was not sufficiently thick to prevent contact between the working surfaces. Thus, the influence of surface roughness on the pressure distribution became significant. Large pressures were generated in the interaction regions of the most prominent surface asperities.

Kondratiuk and Kuhn (2011) analyzed hot-dip-aluminized and electro-plated Zn–Ni coatings on manganese boron flat steel for hot forming applications [7]. The coatings’ tribological behavior in hot strip drawing tests was examined. The experiments were conducted with two different loads and tool geometries and included obtaining the coefficient of friction and wear characteristics.

Ghiotti and Bruschi (2010) considered the tribological behavior of diamond-like carbon (DLC) coatings for sheet metal forming tools [8]. Improper lubrication policies may have a negative impact on the environment. The reason is the use of unhealthy degreasing agents to wash the formed parts. The test results show that in lubricated conditions, the friction coefficient was not significantly influenced by different coatings. The DLC coatings exhibited low friction values in dry conditions only.

Lee et al. (2002) proposed a new model of friction caused by lubrication and surface roughness in sheet metal forming [9]. The experimental results were obtained on a manufactured friction tester. The objective was to find the effect of the lubricant’s material properties and viscosity on the frictional characteristics of both coated and uncoated metals. The friction coefficient was inversely proportional to lubricant viscosity. The FEM analysis with the authors’ model more accurately approximated the experimental results than the FEM analysis using the conventional friction model.

Kirkhorn et al. (2013) studied the influence of tool steel microstructure on friction in sheet metal forming [10]. They used several tooling materials with high-strength uncoated sheet material as a reference sheet material. The authors constructed a tribo-tester, based on flat-die strip drawing, characterized by full control of the applied normal force and the drawing velocity. The tested tools had extremely diverse microstructures. The variation in carbide content could not be directly correlated to variation in the friction coefficient.

Aleksandrović et al. (2011) presented experimental results on the investigation of a specific tribological system’s influence on the non-monotonous two-phase deep drawing process of low-carbon electro-galvanized steel sheets [11]. The first phase involved uniaxial tension in the strips until the elongation reached 10% of producing the blank. This was followed by the deep drawing. The drawing force and distribution of the main strains in the sheet plane were monitored. The authors stated that it was possible to use the concrete non-monotonousness method of forming to improve the process results.

Novotny et al. (2022) analyzed a new (composite) coating for deep drawing tools [14]. It consisted of micro-layers of TiAlN and TiAlCN, applied using high-power impulse magnetron sputtering (HIPIMS) coating technology. The drawing tool for the production of cartridges was made of STN 14109 steel. The objective was to relate the thickness of the layers and their connectivity with the underlying substrate. The authors found that this micro-coating, at a thickness of 5.8 μm, increased the repeatability of production strokes by 200%. This was confirmed by testing in real operation by a large manufacturing company.

Radwanski et al. (2021) analyzed the impact of the stretch leveling process of DC03 and DC04 steel sheets on their quality [15], meaning the waviness and state of internal stresses of the sheets. The achieved reduction values in sheet waviness were 88% and 96% in the cases of the DC03 and DC04 thin sheets, respectively. The residual stresses, after straightening, did not exceed 40 MPa. Thus, stretch leveling with a controlled elongation value resulted in a favorable and stable stress state in the sheets.

Szewczyk et al. (2022) considered the frictional characteristics of deep drawing quality steel sheets in the flat die strip drawing test of 0.8 mm thick DC04 steel sheets [16]. They conducted friction tests under different pressure and lubrication conditions. In the dry friction conditions, the average and the root mean square roughness decreased (in the normal pressure range of 3–6 MPa). Then, they increased due to ploughing mechanism intensification. The use of engine oil decreased the COF values only by 3.84 to 8.87%. The use of 80W-90 gear oil caused decreases in these values by 11.24 to 15.7%.

Dixit (2020) conducted a review of the metal forming modeling methods of various metal forming processes [17]. He emphasized that modeling micro- and nano-forming is quite different from modeling conventional metal forming processes. The scale effect comes into play, while the physical phenomena, which are insignificant at the macro-scale, could become significant at the micro- and nano-scales. Dixit concluded that fairly accurate models are available for predicting the forming load. However, that is not the case for residual stresses and surface integrity, so “the multiscale modeling of the metal forming process may be a viable panacea in future”.

The objective of Gill et al. (2016) was to show the influence of defining the pressure-dependent friction coefficient on numerical spring-back predictions of three steels [18]. The pressure-dependent friction models of each material were compared to the experimental results of a strip drawing test and used in the numerical simulation of an industrial automotive part drawing process. The results show important differences between defining a pressure-dependent or a constant friction coefficient.

Drossel et al. (2019) constructed a novel mechatronic system for measuring and controlling the normal force distribution in deep drawing based on the force measuring platform between the upper die and the press ram [19]. The systematic adjustment and measurement of the resulting force location present the new possibility of controlling the drawing process, as well as ensuring process reliability and drawn part quality.

Tiwari et al. (2022) conducted a review of studies on factors affecting the deep drawing process, including friction, blank holder force, lubrication, process temperature and the drawn part’s shape [20]. They stated that by optimizing the said factors’ influence on the process, one can predict the process’ results, i.e., obtain the required product without compromising its quality.

Ikumapayi et al. (2022) performed “a concise overview of deep drawing”, covering the process applications, merits and demerits of the deep drawing process [21]. The authors stated that there is a scarcity of information on the metallurgical features of warm deep drawing.

Hetz et al. (2020) considered so-called “spring-back” behavior in cross-profile deep drawing [22]. Such a behavior is a consequence of residual stresses that appear in semi-finished product. The authors proposed a novel approach to investigate the spring-back behavior of AA7020-T6 and AA7075-T6 via the spring-back angle. They also advised that it is important to study spring-back behavior at elevated temperatures.

Ma et al. (2015) examined the effect of the friction coefficient on the deep drawing of aluminum alloy AA6111 under three conditions of elevated temperature using finite element analysis and experimental investigation [23]. Their results indicate that the friction coefficient and lubrication position significantly influence the minimum thickness of the drawn piece, as well as its thickness deviation and the failure mode. They concluded that when the friction coefficient is 0.15, the formability is acceptable.

Dwivedi and Agnihotri (2017) conducted a study of deep drawing process parameters with the objective of optimizing the deep drawing process [24]. The considered parameters included blank holding force, friction and blank holder pressure. The authors concluded that for a successful deep drawing manufacturing process, a deep knowledge of all the parameters affecting the process “is a must”.

This review of the literature shows that different deep drawing processes have been studied from various points of view. The considerations and presented results are relevant and useful for improvement of this processing method.

The phenomenon of friction in deep drawing procedures is a significant factor for the successful development of the process without defects in the structure. The basis of the research presented in this paper is the possibility of controlling the friction process for the duration of the process while simultaneously applying a TiN coating to the tool.

The papers analyzed in this section indicate that the research in this direction is relevant.

## 3. Materials and Methods

**Figure 1:**
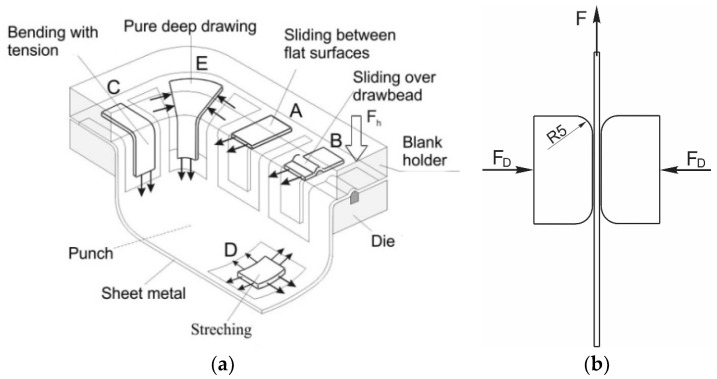
(**a**) Scheme of the physical model of a part with complex geometry; (**b**) scheme of the flat die test.

The deep drawing of parts with complex shapes implies the existence of various influential parameters, making it one of the most difficult and demanding forming processes. Thus, the complete tribological modeling of such a forming process must be based on the principle of physical modeling. Figure 1a shows the physical model of a part with complex geometry, while Figure 1b presents a scheme of the flat die test (model “A” of Figure 1a—thin sheet sliding (strip pulling) between the flat surfaces of the holder and the die) [25]. Model “A” corresponds to the zones of the complex part that are not subjected to lateral compression, just to stretching in the radial direction. The drawing force, as a consequence of the pulling action, is transferred by the die’s edges rounding to the zones below the holder. Due to the fact that the surface pressure during the sliding remains lower than the yield strength, the deformation is within limits that would guarantee that no cracks or any other type of damage would occur. Irregularities in the contact surfaces due to wear or even glued particles can disturb the stable course of the sliding process, and damage or failure of the drawn part can occur [25,26].

### 3.1. The Experimental Apparatus and Setup

**Figure 2:**
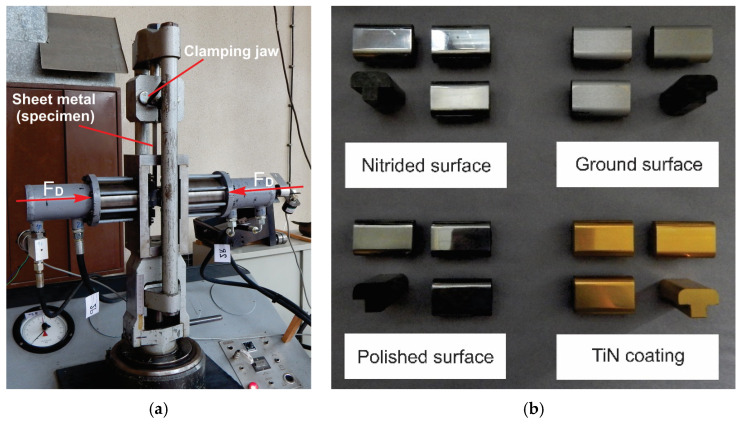
(**a**) Mechanical part of the apparatus during pulling; (**b**) exchangeable sliding elements [27,28].

**Figure 3:**
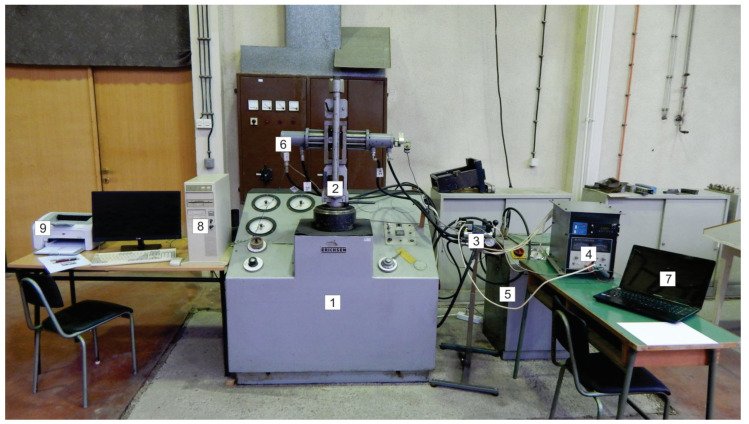
System for passing sheet metal strip between the flat contact surfaces.

**Figure 4:**
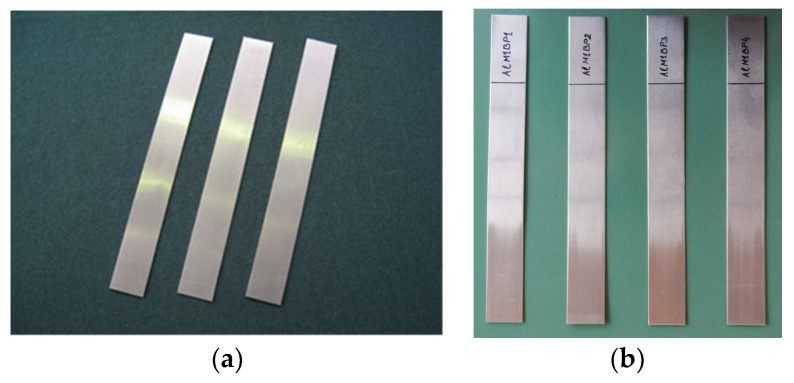
Photographs of the sheet strip samples of AlMg4.5Mn0.5 (**a**) before the drawing process; (**b**) after the drawing process.

The originally designed experimental apparatus used in this experimental investigation is shown in Figure 2a, while its exchangeable sliding elements are shown in detail in Figure 2b. A detailed description of the device parts, including the micro-control unit and the separate hydraulic module (the voltage proportional valve), is given in Figure 3 [27]. For a certain value of voltage signal from the control card, obtained from the controller, a certain flow, i.e., a certain pressure in the cylinder, is obtained, which ensures the blank holding force. The force is transferred to the exchangeable elements (made of the alloyed tool steel X37CrMoV5-1), which hold the thin sheet strip of AlMg4.5Mn0.5 (Figure 4). Since the surfaces are different, the surface roughness values of the elements were determined as well (Table 1) [28].

**Figure 5:**
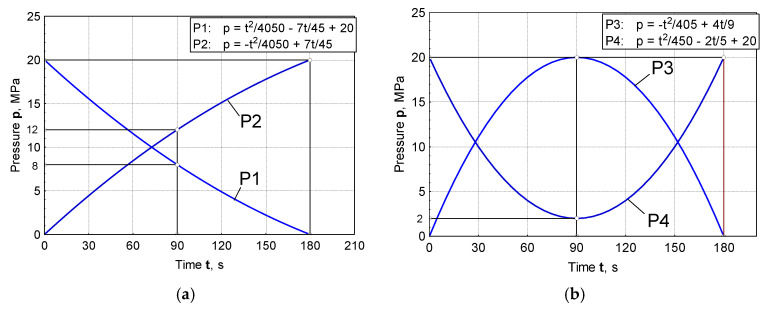
The pre-defined pressure functions: (**a**) P1 and P2; (**b**) P3 and P4.

Prior to the start of the test, it was necessary to determine and define four dependencies of the contact pressure according to which the experiment was to be performed. Those non-linear functions (P1 to P4), presented in Figure 5a,b, were defined based on empirical values of the minimum and maximum pressure (0 to 20 MPa) [28,29]. The pressure variation step is 60 mm, corresponding to the laboratory press properties [30].

Figure 3 shows a photograph of the original apparatus that was used to apply variable pressure between the contact elements and Al alloy sheet samples.

The structure of the system (Figure 3) consists of: (1) a laboratory hydraulic press with triple action, the role of which is to provide the main pulling stroke and realize the traction force; (2) assembly of the mechanical–hydraulic part of the device for obtaining variable contact pressure with replaceable contact elements (Figure 2a); (3) the hydraulic module, which consists of the proportional valves and a distributor; (4) a module for program management of the hydraulic system, to provide the predetermined functional dependencies of pressures, simultaneously, for the duration of the process; (5) a hydraulic unit for powering the press; (6) the actual pressure transmitter; (7) a computer for creating and memorizing programs; (8) a computer with modules for measuring the traction force and actual contact pressure; (9) a printer for printing results.

In the experiment, strips of an AlMg4.5Mn0.6 alloy sheet were used, dimensions 250 × 30 × 0.9 mm (Figure 4), using the two types of lubricants: oil for deep drawing and lubricating grease based on MoS_2_.

The flattening (thinning) of the sheet in this test was negligible, so a sheet thickness of 0.9 mm was considered approximately unchanged during the experiment. Deformation in the direction of sheet thickness, according to model A (Figure 1a), is not significant for the sheet sliding process between flat contact surfaces [29]. The selected aluminum alloy has very good mechanical properties (especially strength), and it is suitable for the production of certain parts of the car body due to its very good corrosion resistance and relatively low specific weight. The chemical composition of the alloy is given in Table 2.

Deep drawing oil was used as one of the lubricants, containing appropriate amounts of refined mineral oil, corrosion inhibitors and additives (compounds based on phosphorus and inactive sulfur). It is used mainly in the automotive industry in a concentrated form or as a pseudo-emulsion with water (water is gradually added to the oil with stirring). It is applied to the processed objects with a brush or a sponge, while it is removed with industrial degreasing agents. The second type of lubricant was a grease based on MoS_2_, and it is a homogeneous dispersion of lithium soaps of higher fatty acids in refined mineral oil. It contains a special group of additives to increase the resistance of the lubricating layer to high pressures and molybdenum disulfide powder. The grease is dark gray in color and has good mechanical, oxidation and thermal stability at elevated temperatures. It has a high bearing capacity and lubricating layer resistance. It enables the reduction of wear even in harsh lubrication conditions. It is in accordance with the ISO 2176:1995/Cor 1:2001 standard, [31].

A significant number of tests were conducted. One sample of sheet metal strip was used for each of the test conditions, which include: type of lubricant, type of contact surfaces of sliding elements, function of pressure change (P1 to P4, Figure 5a,b). The output parameters were diagrams of traction forces and actual contact pressures. The friction coefficients were calculated for each of the mentioned conditions based on the obtained values of traction forces, actual contact pressures and the known contact area between the sheet metal strip and the sliding elements. Diagrams of the actual contact pressures are presented in detail in [28].

### 3.2. Previously Defined Pressure Dependencies

Four variable dependencies of the contact pressure in terms of time were predefined for the needs of the planned experiment [28].

The general form of the quadratic function is given by expression:

(1) $$p=a\cdot t^{2}+b\cdot t+c,$$

where *a*, *b* and *c* are the unknown constants. For the pressure curve P1 (Figure 5a), the constants were determined from the following conditions:

At *p* = 20 MPa and at *t* = 0, expression (1) gives

*c* = 20. (2)

At *p* = 0 MPa and at *t* = 180 s, expression (1) gives

0 = 32,400 *a* + 180 *b* + 20. (3)

At *p* = 8 MPa and at *t* = 90 s, expression (1) gives

8 = 8100 *a* + 90 *b* + 20. (4)

Based on expressions (3) and (4), one solves for the other two unknown constants:

(5) $$a=\frac{1}{4050}\;;\;b=-\frac{7}{45}\;$$

and obtains the expression for the pressure variation function P1 as

(6) $$p=\frac{1}{4050}t^{2}-\frac{7}{45}t+20\;.$$

In a similar way, it was possible to define the functional dependencies for the remaining three pressure changes (P2, P3 and P4, Figure 5a,b), Table 3.

## 4. Results and Discussion

**Figure 6:**
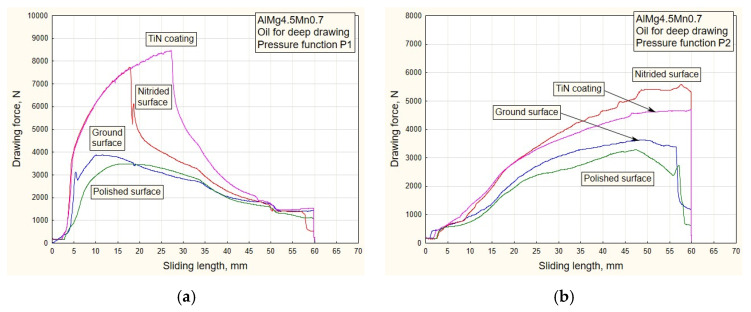
Experimental dependencies of drawing forces for all types of contact surfaces using the deep drawing oil and pressure functions: (**a**) P1; (**b**) P2.

**Figure 7:**
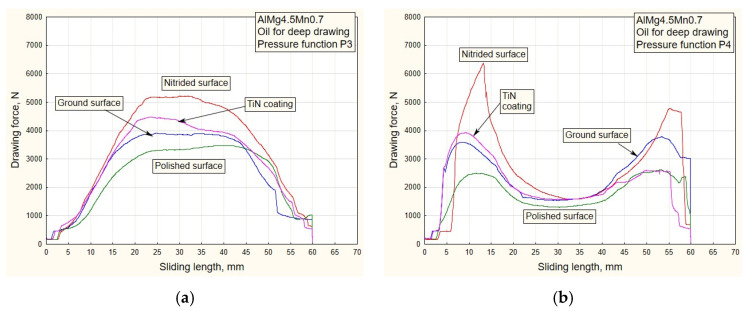
Experimental dependencies of drawing forces for all types of contact surfaces using the deep drawing oil and pressure functions: (**a**) P3; (**b**) P4.

**Figure 8:**
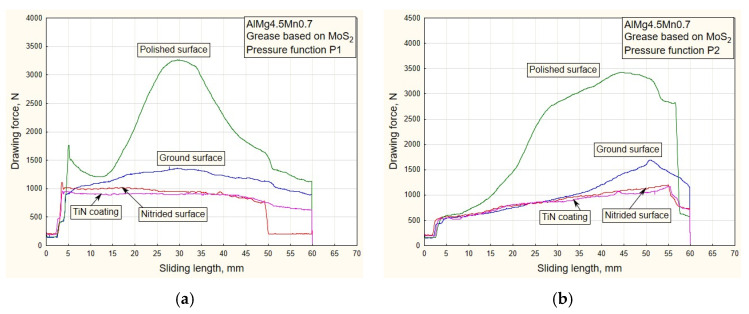
Experimental dependencies of drawing forces for all types of contact surfaces using MoS_2_-based lubricant and pressure functions: (**a**) P1; (**b**) P2.

**Figure 9:**
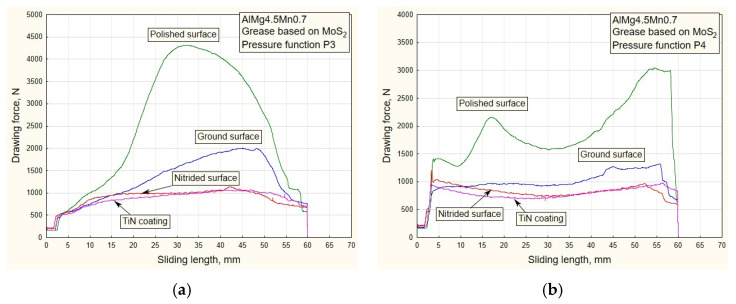
Experimental dependencies of drawing forces for all types of contact surfaces using MoS_2_-based lubricant and pressure functions: (**a**) P3; (**b**) P4.

Figure 6, Figure 7, Figure 8 and Figure 9 show the dependencies of drawing forces for samples of the Al alloy sheet AlMg4.5Mn0.7.

The values of the drawing forces and actual pressures were determined, for each specific case, on a stroke of a length of 60 mm.

The influence of the decreasing variation P1 (Figure 5a) and the high initial value of the pressure while using the oil for deep drawing manifested as an increase in tensile stress (difficult sliding) for surfaces of higher roughness, i.e., nitrided surfaces and those with TiN coating (Table 1). Somewhat lower values of drawing force were obtained by applying the increasing pressure variation P2 (Figure 6b). In the diagrams of Figure 7 (pressure variations P3 and P4), the trend of drawing force dependence is in accordance with the character of the predetermined pressure variations (Figure 5b). Roughness has the greatest influence on the drawing force.

With the use of lubricants based on molybdenum disulfide, there is a phenomenon of increasing drawing force values for surfaces with the least roughness, that is, polished surfaces (Figure 8 and Figure 9).

Al alloy sheets are much softer than steel sheets [26] and have an increased affinity for the formation of “stickers” (glued material) on the contact surfaces of sliding elements, especially those of less roughness. This results in an increase in drawing force with lubricants of higher density. Another type of surface with lesser roughness is polished surfaces (Table 1). There is a noticeable increase in drawing force (Figure 8a,b) with regard to the application of oil for deep drawing (Figure 6 and Figure 7). For surfaces of lesser roughness, the lubricant of higher density (MoS_2_) makes it more difficult to stay in the contact zone. This is an advantage for surfaces of higher roughness (TiN coated and nitrided surfaces), where a sustainable lubricant layer is present during the process. A similar conclusion can be drawn for the pressure variations P3 and P4 (Figure 9a,b).

**Figure 10:**
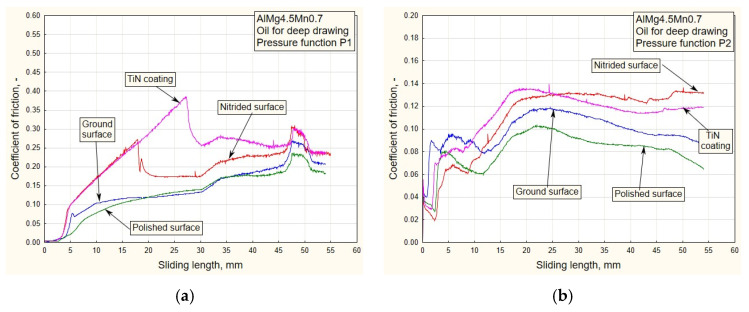
Experimental dependencies of the friction coefficient for all types of contact surface using the deep drawing oil and pressure functions: (**a**) P1; (**b**) P2.

**Figure 11:**
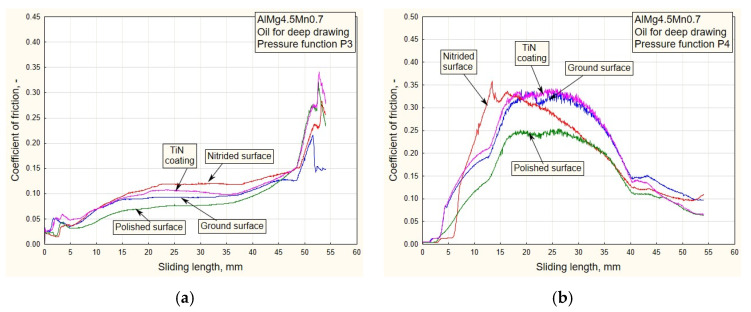
Experimental dependencies of the friction coefficient for all types of contact surface using the deep drawing oil and pressure functions: (**a**) P3; (**b**) P4.

**Figure 12:**
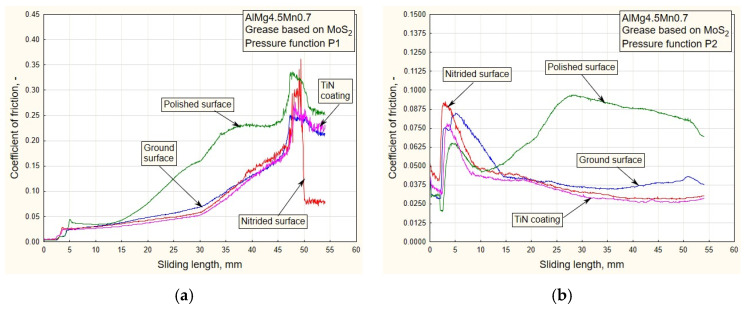
Experimental dependencies of friction coefficients for all types of contact surface using grease based on MoS_2_ and pressure functions: (**a**) P1; (**b**) P2.

**Figure 13:**
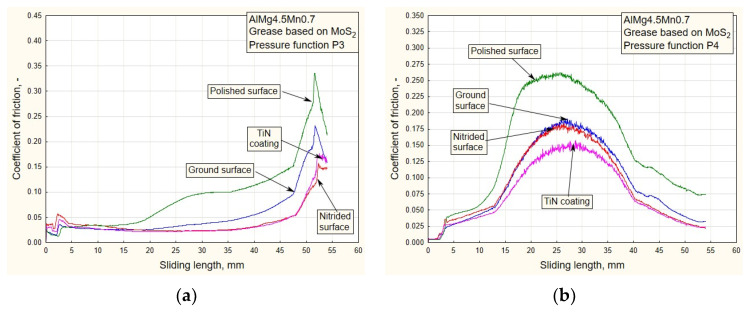
Experimental dependencies of friction coefficients for all the types of contact surfaces using the grease based on MoS_2_ and pressure functions: (**a**) P3; (**b**) P4.

In addition to the drawing force, the coefficient of friction is one of the output parameters of the process by which it is possible to realistically see the influences of variable pressure, surface roughness and the type of lubricant on friction in the sliding zone. In this research, the coefficient of friction was calculated based on the actual (measured) values of drawing forces and the real contact pressure (Equation (7)). Given that the pulling process takes place between the flat contact surfaces (Figure 1b), the coefficient of friction *μ* was easy to determine based on the known value of the actual contact surface of the replaceable elements and sheet metal strips, *A* = 960 mm^2^:

(7) $$\mu =\frac{F}{2\cdot F_{D}}=\frac{F}{2\cdot p_{D}\cdot A}=\frac{F}{1920\cdot p_{D}},$$

where *F* is the strip pulling (traction) force, *F_D_* is the blank holding (contact) force and *p_D_* is the contact pressure. Figure 10, Figure 11, Figure 12 and Figure 13 show the dependencies of the friction coefficient on the sliding stroke length.

By analyzing the diagrams (Figure 10 and Figure 11) for all types of contact surface, a similar conclusion can be drawn as from the drawing force diagram. It is noticeable that the coefficient of friction has lower values for the ground and polished surfaces. The reason for this is a better combination of contact conditions (oil and less rough surfaces, Figure 6 and Figure 7). The type of pressure dependence has a significant influence on the friction coefficient. In the case of decreasing variation (P1, Figure 10a), high pressure values at the beginning of the stroke led to tension and breakage of the sheet samples for surfaces of higher roughness (Table 1). With the increasing change of P2, the pressure gradually increased to the maximum values, so the contact conditions were somewhat more favorable.

In the process of drawing aluminum alloy sheets in combination with lubricating grease based on MoS_2_, there is a problem of sticker formation on contact surfaces of smaller roughness (polished and partially ground). In addition, the viability of the lubricating layer was compromised. On the drawing force diagrams, this was manifested by a disproportionate increase in the drawing force for polished surfaces (Figure 8 and Figure 9). Exactly the same manifestation is represented in the diagram of friction coefficients (Figure 12 and Figure 13).

On the other hand, there is the problem of determining the real values of the friction coefficients for pressure changes that end in a downward trend: P1 and P3 (Figure 12a and Figure 13a). In these cases, it is possible to control the friction until the moment when the pressure drops so much that the contact between the sheet and the sliding elements becomes questionable. These are the values that are close to zero. On the diagrams, this moment is manifested by a sharp jump in the friction coefficient.

## 5. Conclusions

As the starting point of this work, an analysis of the design, manufacture and testing of complex measuring and control equipment was carried out. Our main task was to realize a wide range of pressure variation functions, enabling precise measuring of their influence on the change in drawing force and friction coefficient for different tribological conditions, with an emphasis on titanium nitride coatings. The conclusions can be organized as follows:

(a) In the case of pressure changes that begin with a decreasing trend (P1 and P4 up to half of the stroke, Figure 5a,b), higher values of traction forces and friction coefficients are noticeable, with respect to changes in P2 and, in part, P3. High values of contact pressure at the beginning of the stroke adversely affect the retaining of the lubricant in contact. Applying the functional change P2 (Figure 5a) results in significantly lower values of traction forces and friction coefficients (Figure 6b, Figure 8b, Figure 10b and Figure 12b), which makes the sliding process significantly easier. With this change, the pressure values at the beginning of the stroke are small and gradually increase to the maximum, which keeps the lubricant in the contact zone longer for the duration of the pulling stroke;
(b) The problem appears to be determining the real values of the friction coefficients for pressure changes that end in a downward trend: P1 and P3 (Figure 11a, Figure 12a and Figure 13a). In these cases, it is possible to control the friction until the moment when the pressure drops so much that the contact between the sheet and the sliding elements becomes questionable. These are the values close to zero. On the diagrams, this moment is manifested by a sharp jump in the coefficient of friction;
(c) The change in P3 (Figure 5b) ends with an intensely decreasing trend (from the middle to the end of the stroke). Thus, considering the tendency of the pressure towards zero, unrealistic values for the friction coefficient were obtained; see expression (1). These are the values on the diagram that have a jumpy trend (Figure 11a). Discarding the pressure values for the part of the stroke from 45 to 55 mm (Figure 11a), the average values of the coefficient of friction, according to the type of surface, are: 0.13—nitrided surfaces, 0.105—TiN coating, 0.09—ground surfaces and 0.075—polished surfaces, which correlates with the diagrams of drawing forces under the same conditions (Figure 7a);
(d) Contact surfaces of different roughness values, in combination with oil or lubricating grease, have different effects on the drawing force and friction coefficient. This is largely influenced by the character of the functional pressure variation. It is more suitable to use surfaces of lesser roughness (ground and polished) in combination with oil, while lubricating grease is recommended in combination with surfaces of higher roughness (nitrided surfaces and TiN coatings, Table 1);
(e) The tendency of surfaces of a lesser roughness (mostly polished) towards formation of glued Al sheet layers was observed in the contact zone. This phenomenon is particularly prominent when lubricating was performed with grease based on MoS_2_, especially in conditions of high contact pressures at the beginning of the stroke of the decreasing pressure changes P1 and P4 (Figure 8b and Figure 9b). The reason for this is that the lubricant is then partially pushed out of the contact zone. On the other hand, the nitrided surfaces and titanium nitride coatings had a better ability to retain MoS_2_-based lubricant in the surface roughness depressions (Figure 12a and Figure 13b). A lower affinity of the polished and ground surfaces towards the formation of glued patches was present when lubrication was performed with oil (Figure 10a and Figure 11b);
(f) Titanium nitride coatings, which are mainly used in metal cutting, are suitable for use in sheet metal forming by deep drawing in combination with lubricating greases based on MoS_2_ under all pressure change conditions (Figure 8, Figure 9, Figure 12 and Figure 13) due to their better ability to retain lubricant in surface roughness depressions, even at higher pressures;
(g) Better retention of the lubricating grease in surface roughness depressions was observed with the TiN coating, which makes the lubricating layer sustainable, even at higher contact pressure values. In such conditions, the formation of glued layers of sheet metal on replaceable sliding elements is minimal;
(h) The stability of the TiN coatings of the sliding elements was not affected, even after a significant amount of testing as well as the periodic removal of glued Al alloy sheet layers from the contact surfaces of the coating.

## Figures and Tables

**Table 1:** Surface roughness of the contact elements before the sliding process.

Type of Surface	Surface Roughness, Ra, μm
Nitrided	0.291
TiN coating	0.270
Ground	0.050
Polished	0.038

**Table 2:** Chemical composition of AlMg4.5Mn0.5 alloy.

	Mg	Mn	Si	Fe	Cu	Ti	Zn	Cr
%	4.20	0.57	0.0869	0.29	0.013	0.007	0.068	0.092

**Table 3:** Equations for the pressure variation functions.

Function	Equation	Figure
P1	$p=\frac{t^{2}}{4050}-\frac{7\cdot t}{45}+20$	Figure 5a
P2	$p=-\frac{1t^{2}}{4050}+\frac{7\cdot t}{45}$	Figure 5a
P3	$p=-\frac{t^{2}}{405}+\frac{4\cdot t}{95}$	Figure 5b
P4	$p=\frac{t^{2}}{450}t^{2}-\frac{2\cdot t}{5}+20$	Figure 5b

## Data Availability

Not applicable.
